# Quantified patient preferences for lifestyle intervention programs for diabetes prevention*—*a protocol for a systematic review

**DOI:** 10.1186/s13643-018-0884-5

**Published:** 2018-11-29

**Authors:** Charalabos-Markos Dintsios, Nadja Chernyak, Benjamin Grehl, Andrea Icks

**Affiliations:** 1 Institute for Health Services Research and Health Economics, Centre for Health and Society, Faculty of Medicine, Heinrich-Heine University Düsseldorf, Moorenstr. 5, 40255 Düsseldorf, Germany; 2 Institute for Health Services Research and Health Economics, German Diabetes Center (DDZ), Leibniz Center for Diabetes Research at Heinrich Heine University Düsseldorf, Düsseldorf, Germany; 3 German Center for Diabetes Research (DZD), Neuherberg, Germany

**Keywords:** Diabetes mellitus, Patient preferences, Preferences weights, Preference elicitation, Conjoint analysis, Discrete choice analysis, Analytic hierarchy process, Multi-criteria decision analysis, Systematic review

## Abstract

**Background:**

The 20–70% participation of diabetes patients in lifestyle interventions (LSI) worldwide seems to be rather sub-optimal, in spite of all intents of such interventions to delay further progress of the disease. Positive effects through LSI are expected in particular for patients who suffer less from diabetes-related limitations or other chronic diseases. Seeing that diabetes prevalence and with it mortality are increasing, LSI have become an inherent part of diabetes treatment standards. Various qualitative studies have been carried out to identify participation barriers for LSI. However, these have not resulted in more detailed knowledge about the relative importance of factors with an inhibiting impact on participation. Since it cannot be assumed that all of the influencing factors have equivalent values, it is necessary to investigate their individual importance with regard to a positive or negative decision about participating. There are no systematic reviews on patient preferences for LSI programs in diabetes prevention. As a result, the main objectives of this systematic review are to (i) identify existing patient preference elicitation studies related to LSI for diabetic patients, (ii) summarize the methods applied and findings, and (iii) appraise the reporting and methodological quality of such studies.

**Methods:**

We will perform systematic literature searches to identify suitable studies from 14 electronic databases. Retrieved study records will be included based on predefined eligibility criteria as defined in this protocol. We will run abstract and full-text screenings and then extract data from all selected studies by filling in a predefined data extraction spreadsheet. We will undertake a descriptive, narrative synthesis of findings to address the study objectives, since no pooling for quantified preferences is for methodological reasons implementable. We will pay special attention to aspects of methodological quality of preference elicitation by applying established evaluation criteria of the ISPOR and some own developed criteria for different elicitation techniques. All critical stages within the screening, data extraction, and synthesis processes will be conducted by two pairs of authors. This protocol adheres to PRISMA and PRISMA-P standards.

**Discussion:**

The proposed systematic review will provide an overview of the methods used and current practice in the elicitation and quantification of patients’ preferences for diabetes prevention lifestyle interventions. Furthermore, the methodological quality of the identified studies will be appraised as well.

**Systematic review registration:**

PROSPERO CRD42018086988

**Electronic supplementary material:**

The online version of this article (10.1186/s13643-018-0884-5) contains supplementary material, which is available to authorized users.

## Background

### Disease burden

Diabetes mellitus is a highly prevalent chronic disease affecting approximately 9 to 10% of the global adult population [1]. Based on estimates of the International Diabetes Federation, there were 425 million adults with diabetes in 2017, and this number is expected to rise to 629 million by 2045 [2]. Despite several efforts to improve diabetes care, the disease is still associated with markedly increased morbidity and mortality as well as high cost for the healthcare system [2–5]. The treatment of diabetes mellitus as a chronic disease depends on patient willingness to adopt changes in lifestyle, nutrition, and therapy self-management [6].

### Lifestyle interventions

Lifestyle interventions (LSI) are a central part of diabetes care, in particular in type 2 diabetes mellitus [7–9]. It has been shown that LSI can improve HbA1c and blood pressure [10], slow down the progression of the disease and reduce late complications [11–14], or increase quality of life even if not showing significant long-term differences in cardiovascular morbidity and mortality [15]. The beneficious effect of LSI has been verified, particularly for physical activity, in prediabetes as well, as shown in a recent meta-analysis, improving oral glucose tolerance (risk ratio [RR] − 0.26, 95% CI − 0.06 to 0.07) and fasting blood glucose (RR − 0.05, 95% CI − 0.14 to 0.04) [16]. Furthermore, LSI can added to usual medical care even lead to modest cost savings [17]. Program intensity seems to play a major role in weight loss outcomes, but programs that have high uptake can still have considerable impact in lowering diabetes risk in a population, even with a low-intensity intervention [18]. Furthermore, it seems that LSI are readily scalable and exportable in primary care with potential for substantial clinical and public health impact [19]. Positive effects through LSI are expected in particular for patients between the ages of 35 and 65 years who, in comparison to older patients, suffer less from diabetes-related limitations or other chronic diseases [20]. For this reason, LSI are directed particularly at this age group. In an umbrella review on good practice characteristics of diet and physical activity interventions and policies, the use of theory, participants, target behavior, content development/management, multidimensionality, practitioners, and settings were identified as being relevant for the intervention; participation processes, training for practitioners, the use/integration of existing resources, feasibility, maintenance/sustainability, implementation partnerships, implementation consistency/adaptation processes, and transferability were the important implementation characteristics [21]. However, a 20–70% participation in LSI in general is low [22–24]. Evidence on the impact of incentives to promote LSI remains sparse or as exemplarily shown for physical activity prescriptions of low methodological quality [25].

The choice of particular clinical goals and selection of lifestyle and pharmacologic treatments to reach those goals is a multifaceted process that requires using valid and reliable methods that transparently report on data elicitation processes, potential strengths and shortcomings, and efficiently eliciting patient preferences and values as well by integrating these with evidence of treatment effectiveness as well as provider preferences [26]. Various qualitative studies have been carried out to identify participation barriers for LSI, such as costs and small portion sizes in diet, missing support of family and private issues, strict diet being similarly burdensome to insulin intake, reduced quality of life for dietary interventions, risk of hypoglycemia, work schedule, fear of being tired, low fitness level for physical activity interventions, and finally, psychological distress in general [27–31]. However, these have not resulted in more detailed knowledge about the relative importance of factors with an inhibiting impact on participation. Since it cannot be assumed that all of the influencing factors have equivalent values, it is necessary to investigate their individual importance with regard to a positive or negative decision about participating. In the past, it has been possible to determine the individual importance of various factors relating to participating in such interventions but with a different target population [32, 33]. This raises the question about the quantification of patient preferences for LSI programs in diabetes prevention, especially considering that individual preferences for changes over a LSI are linked with respective outcomes [33]. Tailoring programs rather than using a generic “one size-fits-all” approach should be implemented given some of the observed differences within demographic groups, social-cognitive and behavior constructs with physical activity counseling, and programming preferences. Meeting the needs, interests, motivation, and level of behavior may help to increase physical activity adherence over time. This means developing specific programs for a variety of settings, intensities, structures, and types of activity [31].

### Preferences

Preferences are comparative judgements between entities [34]. In this sense, a preference is the choice of one thing over another with the anticipation that the choice will result in greater value, satisfaction, capability, or improved performance of the individual, the organization, or the society. From a patient perspective, preference instruments reveal what is relevant to the patient and can support decisions, which directly or indirectly affect the patient. They may include even non-health outcomes of health interventions [35]. Interestingly, patient preferences can differ compared to physicians’ judgment, as shown in different systematic reviews [36, 37]. Most studies revealed a disparity between the preferences of actual patients and those of physicians. For most conditions, physicians underestimated the impact of intervention characteristics on patients’ decision-making. Especially for type 2 diabetes mellitus (T2DM) medication treatment, there seems to be a high preference agreement for the main health benefits, but the order between patients and physicians differed [38]. But even transferred to the research agenda, views of diabetic patients and their relatives regarding their preferred research fields may differ when compared to current scientific activity in diabetology, leading to a plea to involve diabetic patients and their relatives in the weighting and selection of research topics more often [39, 40].

Health preference research is connected with the following assumptions [41]: (i) it can be used to elicit patient preferences on intervention attributes, and the resulting preference estimates are valid and reliable; (ii) it can be used to improve the design and implementation of health; (iii) designing health interventions to better meet the needs of patients not only improves patient satisfaction, but also improves patient behaviors such as uptake and adherence; and finally (iv) improvements in patient satisfaction, uptake, and adherence as a result of health preference research lead to better health and, ultimately, to more efficient healthcare systems interventions. Since patient welfare is presumably an objective of providing healthcare services, patient values should according to Mühlbacher and Johnson [42] logically play a central role in approval, utilization, reimbursement, and pricing decisions. To give patients’ preferences appropriate roles in the treatment life cycle, a public–private research initiative has recently been launched [43]. Considering patients’ preferences in individual decisions concerning prevention, diagnostics, or therapy has become more and more important (e.g., shared decision-making). Especially, diabetes treatment requires patients to have significant participation in their care and self-management to achieve glycemic control [44]. It has been shown that this may lead to higher satisfaction with the communication and higher acceptance of and adherence to therapy and, thereby, even to improved health outcomes [41, 45, 46].

### Preference elicitation

In a systematic review to identify the use of preference elicitation methods in healthcare decision-making [47], studies applied different elicitation methods with various degrees of complexity. Among others, multi-criteria decision analysis (MCDA) has been used to quantify and capture patients’ preferences [48].

The International Society for Pharmacoeconomics and Outcomes Research (ISPOR) adopted a broad approach for MCDA, including in the consideration of MCDA methods that help deliberative discussions using explicitly defined criteria. A pragmatic definition is given by Mühlbacher and Kaczynski [49]: “MCDA can be seen as a tool to incorporate objective and subjective measures in a transparent decision-making process that identifies and weighs multiple evaluation criteria in order to prioritize different treatment strategies or health technologies.” The value of MCDA lies not only in the results it generates, but also in its capacity to display the logical connection between inputs (i.e., data and assumptions) and outputs in the form of valued consequences (and costs). A MCDA should seek to model as simple as possible, yet sufficiently complex to incorporate all relevant aspects and their values. In general, value measurement, outranking, and goal programming are used in practice as MCDA approach implementing different techniques for decision support. Two reviews show that MCDA has been applied to a broad range of areas in the health care with the use of a variety of methodological approaches [50, 51]. The authors conclude that further research on MCDA techniques should include the development of guidelines on the assumptions underlying different approaches and their practical implications for decision-makers, and further testing of the impact of different MCDA approaches on decision-making. Simplicity and transparency of a MCDA are an aid to understanding the conclusion for decision-makers and other potential users of the analysis. This is challenging when exploring the complexity of MCDA techniques such as conjoint analysis (CA) or discrete-choice experiments (DCE), analytic hierarchy process (AHP), best-worst scaling (BWS), point allocation, swing weights approach, and other defined scales. Of these techniques, DCE is a widely accepted approach to eliciting stated preferences for health outcomes and processes. The important advantage of choice experiments is their foundation in microeconomic utility theory [52]. The use of DCE in healthcare continues to grow dramatically, as does the scope of applications across an expanding range of countries [53] accompanied by a shift towards statistically more efficient designs and flexible econometric models [54]. Furthermore, DCE can produce reasonable predictions of health-related behaviors [55] and has shown a high predictive value for actual behavior in a public health setting [56]. AHP seems also to play a more important role in medical and healthcare decision-making [57] and has been especially used to elicit patient preferences [58, 59] as well as for medication decision-making in T2DM with experts (Additional file 1)[60].

### Preference studies

A preceded feasibility search determined a plethora of studies and some scoping or systematic reviews concerning patient preferences in connection with various medication interventions for diabetes disease [61–67]. It should be noted that there are plenty of available pharmaceutical therapies for diabetes [68]. Yet, in the feasibility search, there were identified a few studies that had been conducted specifically on patient preferences for LSI [69, 70]. Apart from the intervention-relevant influence factors with regard to participating in or designing LSI, these survey studies on patient preferences have also partly examined the willingness to pay and the design of the financial incentives for participating in the programs offered. Each study made reference to the corresponding national healthcare context.

### Study objective and rationale

While there are systematic reviews regarding treatment preferences in people with diabetes, a systematic review adressing LSI preferences is mising. The main objective of this systematic literature review is to identify existing studies eliciting patient preferences for LSI programs for diabetes prevention, summarize the methods applied and findings, and appraise their methodological quality. We will review identified studies with regard to their methodical quality to provide an overview of current practices in patient preference elicitation for LSI programs in diabetes prevention reported in the literature and to find out which program aspects are included and how important they are for surveyed patients and professionals. Since pooling preferences elicited with different methods in various samples is not applicable, we will summarize the findings of the identified studies by narrative comparisons.

## Methods

### Protocol

This protocol adheres to the Preferred Reporting Items in Systematic Reviews and Meta-analyses (PRISMA) statement [71] and PRISMA for systematic review protocols (PRISMA-P) statement [72]. The PRISMA-P checklist is given in Additional file 2. The protocol is registered in the International Prospective Register of Systematic Reviews (PROSPERO) CRD42018086988.

### Eligibility criteria

We will include studies reporting quantified elicitation of preferences on lifestyle programs for diabetes prevention with characteristics given in Table 1. We will exclude studies considering only qualitative and not quantitative preference elicitation. Qualitative approaches follow their own methodology. Their identification and the appraisal of the implemented methods within qualitative studies constitute an own topic in itself which should be captured in a specific systematic review.

**Table 1:** Eligibility criteria in study selection

Criteria	Inclusion	Exclusion
Population	• Patients	• Patients suffering from other conditions than diabetes • Professionals (physicians, nurses, etc.)
Condition	• T1DM and T2DM • All stages of diabetes disease • Complications of diabetes • Metabolic disorders in relation with diabetes	• Conditions unrelated to diabetes
Intervention	• Quantitative elicitation of preferences for lifestyle interventions • All available quantitative methods for stated preferences (MCDA, MADM, MODM, CA, DCE, AHP, BWS, WTP, etc.)	• Qualitative elicitation of preferences for lifestyle interventions • Quantitative or qualitative elicitation of preferences exclusively for pharmacological treatment of diabetes • Elicitation of utilities
Study design	• The study is based on single and/or multiple data sets	
Type of publication	• Publications in peer-reviewed journals which can be retrieved through our search approach • Studies, to which full publication is available	• Editorials, comments, newspaper articles, and other forms of popular media • Conference abstracts • Studies, to which no full publication is available
Date	• No restriction	
Language	• No restrictions on language, but available English, French, Spanish, or German abstract is a pre-condition for inclusion	• No English, French, Spanish, or German abstract available

### Information sources

The following literature databases will be searched: PubMed (via NLM), MEDLINE (via Ovid), Journals@Ovid (via Ovid), EMBASE (via Ovid), EconLit (via EBSCOhost), Web of Science (via Thomson Reuters), Science direct (via Elsevier), and PsycINFO (via Ovid).

To identify potential existing systematic reviews with regard to our research topic, we will also search the Cochrane Library (via Wiley) including the NHS CRD databanks Database of Abstracts of Reviews of Effects (DARE), Health Technology Assessment (HTA), and NHS Economic Evaluation Database (NHS EED). We will also include grey literature databases in our search: CINAHL (via EBSCOhost), Catalogue ZB MED (via ZBMed), Current Contents (via ZBMed), DissOnline (via ZBMed), Econis (via ZBMed), and Econbiz (via ZBW) and finally for the German region the “Karlsruher virtueller Katalog” as well.

### Search strategy

We will not restrict the timeframe of the search nor will we apply any search filters [73] to guarantee for high sensitivity irrespectively of the respective specificity as a result of our feasibility search which yielded only two relevant hits. We composed the “primary” bibliographic search strategy according to PubMed search rules, which will be translated to other database syntaxes. We have applied the search strategy to the relevant studies found in the previous feasibility and known in the research field to check for the search strategy’s sensitivity. The details of this search strategy are given in Table 2.

**Table 2:** Search strategy for MEDLINE (via PubMed)

Databank		PubMed (NLM)
Timespan:		Unlimited
Language:		Unrestricted
Date of creation:		09 September 2017
#	Hits	Query
#1	64,223	“DIABETES MELLITUS, TYPE 1”[MESH]
#2	95,093	“DIABETES MELLITUS, TYPE 2”[MESH]
#3	7315	“DIABETES INSIPIDUS”[MESH]
#4	34,268	“INSULIN/ADMINISTRATION AND DOSAGE”[MESH] OR “INSULIN/ECONOMICS”[MESH] OR “INSULIN/ORGANIZATION AND ADMINISTRATION”[MESH] OR “INSULIN/THERAPEUTIC USE”[MESH] OR “INSULIN/THERAPY”[MESH]
#5	256,585	(diabetes [ti] OR diabetic [ti] OR T1DM [ti] OR T2DM [ti] OR NIDDM [ti])
#6	311,637	#1 OR #2 OR #3 OR #4 OR #5
#7	8688	“RISK REDUCTION BEHAVIOR”[MESH]
#8	69,754	“LIFE STYLE”[MESH]
#9	134,304	“EXERCISE”[MESH]
#10	96,110	“SPORTS”[MAJR]
#11	17,011	“REHABILITATION”[MESH:NOEXP]
#12	4582	“COMMUNITY HEALTH PLANNING”[MESH]
#13	142,616	“HEALTH EDUCATION”[MESH]
#14	59,790	“HEALTH PROMOTION”[MESH]
#15	499	change*[ti] AND diabetes[tiab] AND (diet[ti] OR behavior*[ti] OR behaviour*[ti] OR nutrition [ti] OR activity [ti] OR activities [ti] OR training [ti] OR physical [ti] OR athletic [ti])
#16	1,191,618	(lifestyle [tiab] OR life style [tiab] OR behaviour*[ti] OR diet program*[tiab] OR behavior*[ti] OR exercise [ti] OR training program*[tiab] OR preventive strategy [tiab] OR prevention program*[tiab] OR behavioral prevention [tiab] OR behavioral program*[tiab] OR way of life [tiab] OR manner of living [tiab] OR behavioral intervention*[tiab] OR diet [ti] OR behavioral intervention*[tiab] OR community program*[tiab] OR nutrition [ti] OR activity [ti] OR activities [ti] OR training [ti] OR physical [ti] OR athletic [ti])
#17	2,779,775	#7 OR #8 OR #9 OR #10 OR #11 OR #12 OR 13 OR #14 OR #16
#18	47,980	#6 AND #17
#19	48,163	#15 OR #18
#20	17	#19 AND (conjoint [tiab] OR discrete choice [tiab] OR multi-criteria*[tiab] OR multicriteria*[tiab] OR multi-attribute*[tiab] OR multiattribute*[tiab] OR multi-objective*[tiab] OR multiobjective*[tiab] OR multi-alternative*[tiab] OR multialternative*[tiab] OR multiple criteria[tiab] OR multiple attribute*[tiab] OR multiple objective*[tiab] OR multiple alternative*[tiab])
#21	24	#19 AND (stated preference*[tiab] OR willingness to pay [tiab] OR willing to pay [tiab] OR wtp [tiab] OR analytic hierarchy process*[tiab] OR ahp [tiab])
#22	218	#19 AND (decision-analy*[tiab] OR decision-making [tiab] OR patient preference*[tiab] OR patient* prioriti*[tiab] OR (patient [ti] AND prioritizing[ti]) OR elicit* patient*[tiab] OR preference*[ti] OR health-state utilit*[tiab] OR EQ-5D*[ti])
#23	15	#19 AND (health state utilit* OR utility measure*[tiab] OR health state utilit*[tiab] OR utility value*[tiab] OR utility score*[tiab] OR utility-based score*[tiab] OR health state valuation [tiab] OR m-mauf*[tiab])
#24	19	#19 AND (hui2 [tiab] OR hui3 [tiab] OR hui-2 [tiab] OR hui-3 [tiab] OR health utility [tiab] OR health utilities [tiab])
#25	0	#19 AND (part-worth [tiab] OR paired comparisons [tiab] OR pairwise choices [tiab] OR pairwise choice [tiab])
#26	97	#19 AND (decision-analy*[ti] OR decision-making [ti] OR decision support*[ti] OR preference*[ti] OR prioriti*[ti] OR ((choice*[ti] OR elicit*[ti]) AND patient*[ti]))
27	15	#19 AND (best-worse scaling [ti] OR preference weight [ti] OR willingness to accept [ti] OR out-of-pocket[ti] OR mcda [ti] OR trade-off[ti] OR utilities [ti] OR valuation [ti])
28	2	#19 AND (goal programming OR vector optimization [tiab] OR value theory [tiab] OR maut [tiab] OR mavt [tiab] OR promethee [tiab] OR preference ranking organization [tiab] OR method for enrichment evaluations [tiab] OR electre [tiab] OR elimination et choix traduisant la realite [tiab])
#29	0	#19 AND (mental modeling [tiab] OR trade-off analysis [tiab] OR tradeoff analysis [tiab] OR risk-based prioritization [tiab])
#30	0	#19 AND (((gaia[tiab] OR meteor [tiab] OR kepner-tregoe [tiab] OR archimedean [tiab] OR tchebycheff [tiab] OR min-max [tiab] OR step method [tiab] OR interactive multiple goal programming [tiab] OR imgp [tiab] OR priam [tiab] OR scalarizing function [tiab] OR outranking concept [tiab] OR goal point approach*[tiab] OR reference point approach*[tiab] OR concordance index [tiab] OR concordance indices[tiab] OR discordance indices [tiab] OR discordance index [tiab] OR trade-off term*[tiab] OR trade-off terms [tiab] OR voting weight*[tiab] OR fuzzy set theory [tiab] OR fuzzy sets [tiab] OR correspondence analysis [tiab]) AND (decision-making [tiab] OR decision-analy*[tiab]))
#31	1	25,151,503 [pmid]
#32	103	Similar articles for PubMed (Select 25,151,503)
#33	1	25,348,049 [pmid]
#34	103	Similar articles for PubMed (Select 25,348,049)
#35	57	#34 AND (diabetes OR diabetic OR insulin*)
#36	1	24,289,831 [pmid]
#37	188	Similar articles for PubMed (Select 24,289,831)
#38	55	#37 AND (diabetes OR diabetic OR insulin*)
#39	199	#32 OR #35 OR #38
#40	509	#20 OR #21 OR #22 OR #23 OR #24 OR #25 OR #26 OR #27 OR #28 OR #29 OR #30 OR #32 OR #35 OR #38

### Technical tools

The hits of the systematic literature search will be collected and stored in EndNote (Version ×Patient preferences versus physicians7, Thompson Reuters). The abstract and full-text screening will be carried out using the EndNote Surface. During the data extraction, we will fill in our own spreadsheet forms. All information will be shared and be made available within the working group consisting of two tandems.

### Study selection

We will select studies in several steps. First, we will identify all relevant studies by conducting the search in the scientific publication databases listed in the “Information sources” section. We will remove duplicates to reduce the reviewers’ workload associated with the next steps. Next, we will screen titles and abstracts of all references selected so far. Two tandems of researchers will independently review and include or exclude references guided by the eligibility criteria mentioned above. The title and abstract screening will be piloted first within the tandems independently and then between the tandems to adjust the procedure. Any disagreement and inconsistency in the final decisions will be resolved in the further discussion among all researchers involved in the screening. Full-text screening will be the final step within our study selection process. Four independent researchers (the members of the two tandems) will review the full texts of the studies selected in the previous step to collect the final list of studies for data extraction. The decision on inclusion or exclusion will be made on the basis of the eligibility criteria. Any apparent discrepancies appearing during the full-text screening will be resolved by discussion among the researches. The screening process will be reported accurately and in sufficient detail—including the title and abstract screening and the full-text screening—to complete a PRISMA flowchart. The number of the excluded studies accompanied by respective exclusion reasons will be recorded. Flowcharts for included and excluded studies will be provided.

### Additional data source selection

During the title/abstract screening, we will collect systematic literature reviews addressing LSI in diabetes prevention as a potential reference source of interest. After the full-text screening, we will export the reference list of all publications identified for the data extraction. Moreover, we will check forward citations of these publications by tracking them in different citation databases. We will apply PubMed’s “related articles” feature for the same papers to collect the first 10 hits. The restricted number of hits is voluntarily chosen to reduce the workload. All references gathered in this step will be subject to the same steps as described in the “Study selection” section.

### Data extraction

Two reviewers will independently extract data from all included studies after the full-text review by filling in a predefined data extraction spreadsheet. The data extraction spreadsheet was first piloted. The data extraction spreadsheet is provided in addition to the paper documents (Additional file 1). Data to be extracted will be arranged into the following categories:

1. Research question and sample
2. Perspective of preference elicitation
3. Method of preference elicitation
4. Method of preference quantification and authors’ rationale for its choice
5. Criteria, attributes, and values of preferences
6. Subgroup analysis (as pre-specified in the studies)
7. Results of preference quantification

### Quality appraisal

The data extraction sheet contains questions and categories that are related to the methodological quality appraisal of the patient preference elicitation. These parameters are based among others on different ISPOR task forces (ISPOR MCDA Emerging Good Practices [48, 74], ISPOR Conjoint Analysis Good Research Practices [75], ISPOR Good Research Practices for Conjoint Analysis [76], and ISPOR Conjoint Analysis Experimental Design Good Research Practices [77]) and other materials or checklists discussing the quality of preference elicitation methods [54, 61, 78, 79] completed in addition by own quality criteria, especially for AHP. The quality appraisal includes furthermore the assessment of the appropriateness of the chosen method for the specific research question and the subsequently derived criteria or attributes for the implemented preference elicitation. Additionally, it will be checked if preference elicitations are accompanied by qualitative research since validity and reliability of preference estimators are despite methodological elicitation appropriateness still at stake. If information is not provided, we will assign “not applicable,” “not reported,” or “not enough information” to the corresponding questions.

### Data synthesis

Since patient preference elicitation methods are different and the preference measures are largely diverse, we will concise results in a narrative descriptive analysis summarizing them according to the implemented approaches and contrasting the rankings of comparable attributes or criteria. The latter will be done by clustering respective attributes or criteria according to their type and subsequently by comparing the order and the weights of the ranked attribute or criteria clusters.

## Discussion

This protocol describes objectives, methods, and steps of an upcoming systematic review that will include studies on the quantification of patient preferences for LSI in diabetes prevention. To the best of our knowledge, this contribution to the scientific community is novel since no publications are specially targeting the elicitation of patient preferences for lifestyle interventions in diabetes prevention systematically. The objectives are to identify studies eliciting patient preferences for lifestyle intervention programs for diabetes prevention, appraise their methodological quality and their reporting, and finally summarize findings to provide an overview of the current practice in this field.

## Additional files

Additional file 1: Data extraction spreadsheet. (DOCX 23 kb)

Additional file 2: PRISMA-P checklist. (DOCX 37 kb)

## Abbreviations

AHP: Analytic hierarchy process
BWS: Best-worst scaling
CA: Conjoint analysis
CDSR: The Cochrane Database of Systematic Reviews
CENTRAL: Cochrane Central Register of Controlled Trials
CINAHL: Cumulative Index to Nursing and Allied Health Literature
DARE: The Database of Abstracts of Reviews of Effects
DCE: Discrete-choice experiment
DissOnline: Dissertation online
DM: Diabetes mellitus
Econbiz: Academic search portal provided by the German National Library of Economics – Leibniz Information Centre for Economics
Econis: Academic search portal provided by the German National Library of Economics – Leibniz Information Centre for Economics
EconLit: Economic literature search portal provided by the American Economic Association
EMBASE: Excerpta Medica database
HbA1c: Glycated hemoglobin
HTA: Health technology assessment
IDF: International Diabetes Federation
ISPOR: The International Society for Pharmacoeconomics and Outcomes Research
LSI: Lifestyle interventions
MCDA: Multi-criteria decision analysis
MEDLINE: Medical Literature Analysis and Retrieval System Online
NHS CRD: NHS Centre for Reviews and Dissemination
NHS EED: NHS Economic Evaluation Database
NHS: National Health Service
NLM: US National Library of Medicine
PRISMA: The Preferred Reporting Items in Systematic Reviews and Meta-analyses
PRISMA-P: The Preferred Reporting Items in Systematic Reviews and Meta-analyses for Protocols
PROSPERO: The International Prospective Register of Systematic Reviews
PsycINFO: Psychological literature search portal provided by the American Psychological Association
T2DM: Type 2 diabetes mellitus
ZB MED: German Central Library for Medicine
