# Supplementary material for: Quantified patient preferences for lifestyle intervention programs for diabetes prevention—a protocol for a systematic review
Source: Syst Rev. 2018 Nov 29;7:214. doi: 10.1186/s13643-018-0884-5 (PMC6264623; doi:10.1186/s13643-018-0884-5)
Supplement: Supplementary file 1 — Data extraction spreadsheet. (DOCX 23 kb) [file 13643_2018_884_MOESM1_ESM.docx]

**Example Extraction Spreadsheet Analytical Hierarchy Process (AHP)**

Note on filling out the extraction sheet: Folders in bold print have to be filled out. Assistance for filling out the sheet is offered in italics.

| **Extraction sheet – Analytic Hierarchy Process (AHP)** | | | | | | |
| --- | --- | --- | --- | --- | --- | --- |
| **Generic** | | | | | | |
| **Extracting reviewer:** |  | | | | | |
| **Title:** |  | | | | | |
| **Author(s)/Year of**  **publication:** |  | | | | | |
| **Location:** |  | | | | | |
| **Objectives of study:** |  | | | | | |
| **Perspective:** | **Patient** | | | **General population** | **Professionals** | **Proxies** |
| **Description of sample:** | *In case of patients or proxies: type of diabetes, stage of disease, Age, Gender, Duration of disease, actual treatment, Diabetes complications, etc.*  *In case of professionals: Specialty, In- or Out-patient care, etc.* | | | | | |
| **Sample size:** |  | | | | | |
| **Subgroup analysis:** | **Yes/No** | | **If yes, for which subgroups?** | | | |
| **Specific for AHP** | | | | | | |
| **Generation of criteria for AHP:** | | *Systematic Review, Survey of experts, Focus groups, Patient interviews, Clinical guidelines, Combination, Other, not reported* | | | | |
| **Number of hierarchical levels:** | |  | | | | |
| **What is the over-arching target/decision problem? (highest hierarchical level)** | | *Comparative treatment, Intervention, drug from the perspective of…* | | | | |
| **Which alternatives are available? (lowest hierarchical level)** | | Intervention A, B, C… | | | | |
| **Number of of included criteria and sub-criteria:** | |  | | | | |
| **Used criteria/**  **sub-criteria:** | | **WTP/costs:** state attribute(s)  **Risk:**  **Time requirement:**  **Health status/Disease characteristics:**  **Interventions/Characteristics of technologies:**  **Social aspects:**  **Medical care:**  **Other:** | | | | |
| **Choice of levels/ attribute values/ criteria and sub-criteria and their range:** | | *Systematic Review, Survey of experts, Focus groups, Patient interviews, Clinical guidelines, Combination, Other, not reported* | | | | |
| **Number of** **levels/ attribute values/ criteria and sub-criteria per each cluster** | | Ideally between 3 and 5 *(Hummel et al., 2014)* | | | | |
| **Number of pairwise comparisons** | | x-times, not reported | | | | |
| **Which rating scale was used?** | | *9 points AHP scale, linear scale, geometric scale, logarithmic scale, continuous graphic mode, other* | | | | |
| **Were verbal preferences assigned to scale values?** | | *Yes/No* | | | | |
| **Were correlations/ co-linearities or confounding between attributes/ criteria / sub-criteria considered or discussed?** | | *Yes/No*  *If yes, state the respective information* | | | | |
| **Modes of experiment implementation:** | | *Group decisions (consensus or aggregating individual decisions)*  *or*  *directly (in case of high number of alternatives) with qualitative or quantitative intensity scales* | | | | |
| **Order of valuation:** | | *Bottom-up (first value priorities of alternatives and then criteria weights)*  *or*  *Top-down (first estimate criteria weights and then value priorities of alternatives)* | | | | |
| **Validity** | | | | | | |
| **Validity checks:** | | *External, internal (theoretical, Non-satiation, Transitivity, Sen’s expansion and contraction, compensatory decision making), other, not reported* | | | | |
| **Calculation of criteria weights or Eigenvector:** | | *Eigenvector method, Goal Programming, Logarithmic Least Squares Method (LLSM), Least Square technique, additive normalization, fuzzy preference programming methods, other* | | | | |
| **Aggregation of group decision(s):** | | *Geometric mean, arithmetic mean, other* | | | | |
| **Consistency check:** | | *Consistency ratio >0.1, >0.2, not reported*  *In case of exceeding CR:*  *Restructuring of hierarchy, repeated survey asking to revise comparisons, exclusion of inconsistent cases, other* | | | | |
| **Typology of inconsistencies:** | | **Clerical error:**  *Patients who erroneously answer the inverse of what they intend to say – crossing the value on the wrong side of the scale*  **Use of extreme values:**  *Patients use extreme values to support the direction of their preference. However, extreme values should only be used to express an extreme strength of preference, not a preference in general.*  **Related to model structure, criteria/sub-criteria definition:**  *Criteria/sub-criteria in AHP are to be structured in a way that criteria in a cluster at one level are comparable within an order of magnitude.*  *However, for practical reasons (number of comparisons), this might not always be entirely feasible.*  **Lack of information or understanding or lack of concentration/interest:**  *If patients are not really interested in the questionnaire or do not understand the criteria asked about they tend to give random answers, which often leads to high inconsistencies.*  **Inconsistency:**  *Truly intransitive answers, i.e. if a patients values A higher than B and B higher than C, but then C higher than A* | | | | |
| **Is a sensitivity analysis implemented?** | | *Yes/No, not reported*  *With regard to the valuations or the prioritized criteria*  *Rank reversal occurred? Yes/no*  *If yes, which approaches were used to avoid rank reversal (B-G modified AHP, Suppermatrix approach, referenced AHP, “Normalization to minimum entry”, Multiplicative AHP, Change from distributive mode to ideal ode, other)?* | | | | |
| **Further estimation of uncertainty:** | | *Further efforts to check for certainty or robustness of results* | | | | |
| **Software used:** | | *Team Expert Chioce, Decision Lens, HIPIRE 3+, Super Decisions, SelsectPro Decision Support Software, EasyMind, MakeItRational, TransparentChioce, MindDecider Team, other, not reported* | | | | |
| **Use of qualitative methods/Pilots:** | | *Yes/No*  *If yes, for:*  *Generation of criteria/sub-criteria, levels/values/pilots/pre-test of experiment, explanation/feedback with participants for the choice of alternatives/criteria* | | | | |
| **Was a definite interpretation of criteria and used scales ensured?** | | *Yes/No, not reported* | | | | |
| **“Response efficiency”** | | *Were potential measurement errors reported or discussed? Are they to be expected due to:*   - *too many pairwise comparisons; fatigue* - *unclear definitions, heterogeneous interpretations, no guarantee for a definite understanding* - *cognitive limitations of the study population*   *How was dealt with measurement errors?*   - *Overlapping of attribute values in pairwise comparisons* - *other* | | | | |
| **Results** | | | | | | |
| **Main results of the AHP experiment:** | | *Results for the AHP preference elicitation reported by the authors of the study* | | | | |
| **Conclusions/Hypotheses:** | | *Which are the essential discussion points and conclusions – pls. present them divided in clinical/content-relevant and methodical aspects.* | | | | |
| **Limitations and Transferability of results:** | |  | | | | |
| **Miscellaneous:** | | *All relevant/interesting information not covered or captured by the extraction sheet* | | | | |
